# Reduced Anxiety in Forensic Inpatients after a Long-Term Intervention with Atlantic Salmon

**DOI:** 10.3390/nu6125405

**Published:** 2014-11-26

**Authors:** Anita L. Hansen, Gina Olson, Lisbeth Dahl, David Thornton, Bjørn Grung, Ingvild E. Graff, Livar Frøyland, Julian F. Thayer

**Affiliations:** 1 Department of Psychosocial Science, University of Bergen, Christiesgt. 12, 5015 Bergen, Norway; 2 Centre for Research and Education in Forensic Psychiatry, Haukeland University Hospital, 5021 Bergen, Norway; 3 Sand Ridge Secure Treatment Center (SRSTC), P.O. Box 0700, 1111 North Road, Mauston, WI 53948, USA; E-Mails: Gina.Olson@dhs.wisconsin.gov (G.O.); david.thornton@dhs.wisconsin.gov (D.T.); 4 National Institute of Nutrition and Seafood Research (NIFES), P.O. Box 2029, Nordnes, 5817 Bergen, Norway; E-Mails: lisbeth.dahl@nifes.no (L.D.); ingvild.graff@nifes.no (I.E.G.); livar.froyland@nifes.no (L.F.); 5 Department of Chemistry, University of Bergen, Allégaten 41, 5007 Bergen, Norway; E-Mail: bjoern.grung@kj.uib.no; 6 Department of Psychology, the Ohio State University, 1835 Neil Avenue, Columbus, OH 43210, USA; E-Mail: thayer.39@osu.edu

**Keywords:** anxiety, heart rate variability, fatty fish consumption, fatty acids, vitamin D

## Abstract

The aim of the present study was to investigate the effects of Atlantic salmon consumption on underlying biological mechanisms associated with anxiety such as heart rate variability (HRV) and heart rate (HR) as well as a measure of self-reported anxiety. Moreover, these biological and self-reported outcome measures were investigated in relation to specific nutrients; vitamin D status, eicosapentaenoic acid (EPA) and docosahexaenoic acid (DHA). Ninety-five male forensic inpatients were randomly assigned into a Fish (Atlantic salmon three times per week from September to February) or a Control group (alternative meal, e.g., chicken, pork, or beef three times per week during the same period). HRV measured as the root mean square of successive differences (rMSSD), HR, state- and trait-anxiety (STAI), were assessed before (pre-test) and at the end of the 23 weeks dietary intervention period (post-test). The Fish group showed significant improvements in both rMSSD and HR. The Fish group also showed significant decreases in state-anxiety. Finally, there was a positive relationship between rMSSD and vitamin D status. The findings suggest that Atlantic salmon consumption may have an impact on mental health related variables such as underlying mechanisms playing a key role in emotion-regulation and state-anxiety.

## 1. Introduction

Fish consumption is associated with beneficial effects on a number of health outcome variables. The strongest evidence is the prevention of cardiovascular disease [1]. However, there is also emerging evidence related to prenatal development during pregnancy [2], maintenance of cognitive functioning in both children [3] and the elderly [4] as well as mental health in adults [5,6]. However, most of the knowledge concerning the relationship between fish consumption and mental health is based on self-reported dietary patterns and the results seem to be inconclusive [5,7,8].

Fatty fish (e.g., salmon, mackerel, herring) are important dietary sources of marine omega-3 fatty acids (eicosapentaenoic acid; EPA and docosahexaenoic acid; DHA) [9]. Usually the relationship between fish consumption and improved mental health (e.g., reduced negative affect, such as depressive mood) has been explained by these underlying nutrients [5,6,10]. However, by using self-report questionnaires neither Hakkarainen *et al.* [7] nor Schiepers *et al.* [8] found any relationship between fish consumption and mental health measured as depressed mood and mental well-being, respectively. Neither was any relationship between mental health and fatty acid status discovered. Interestingly, Schiepers *et al.* [8] found a positive relationship between fish consumption, but not fatty acids, and physical well-being. However, the results concerning fish consumption and mental health are inconclusive.

Importantly, fatty fish is also a source for other nutrients such as vitamin D, iodine, selenium and protein [9]. Lansdowne and Provost [11] found in a randomized double blind study that vitamin D supplementation during late wintertime enhanced positive affect and reduced negative affect. In a dietary intervention study during wintertime, fatty fish consumption had a positive influence on sleep and daily functioning, and sufficient vitamin D status was positively related to the beneficial effects [12]. The fish group had significantly increased levels of the marine omega-3 fatty acids EPA and DHA. However, the sum EPA + DHA did not correlate with sleep or daily functioning. Thus, in order to establish a causal relationship between fish consumption and mental health, there is a need for experimental dietary intervention studies investigating underlying mechanisms associated with mental health. Since the mechanisms associated with the effects of fish consumption are poorly understood, an identification of the underlying biological markers is needed.

Mozaffarian *et al.* [13] emphasized heart rate variability (HRV) as one potentially useful outcome measure in relation to fish consumption. HRV, which is a measure of the continuous interplay between sympathetic and parasympathetic influences on heart rate (HR), has been shown to be associated with both physical and mental health [14,15]. Thayer and Lane [14] have developed a network model that explains the relationship between psychological processes and physiological underlying structures. In this model they emphasized the role of HRV as an index of emotion-regulation. Importantly, low HRV is one of the physiological characteristics of affective disorders such as generalized anxiety disorder (GAD) and panic disorder (PD) [16,17]. It has recently been reported that low baseline HRV characterizes anxiety across a range of specific disorders including GAD, PD, social anxiety (SA), and obsessive-compulsive disorder (OCD) [18]. Importantly, a recent meta-analysis also demonstrated that different HRV parameters were inversely related with anxiety disorders [19]. Thus, HRV is an indicator of emotion- regulation, and the higher HRV, the more adaptive and flexible the organism is [20,21].

In a population-based study Mozaffarian *et al.* [13] investigated the relationship among self-reported habitual fish intake, omega*-*3 consumption and different measures of HRV. HRV can be operationalized through a variety of measures such as time-domain (e.g., root mean square successive differences; rMSSD), respiratory sinus arrhythmia (RSA) and frequency-domain (high frequency (HF), low frequency (LF) and LF/HF-ratio) [21]. However, in this study Mozaffarian revealed that consumption of tuna or other fish was positively linked to different HRV parameters such as HF, LF/HF and rMSSD when other lifestyle factors were controlled for. On the other hand, Erkkilä *et al.* [22] investigated the effects of a lean or fatty fish intervention for eight weeks on rMSSD HRV in subjects with coronary heart disease. However, the results from this study indicated that the rMSSD HRV did not change significantly in either of the fish groups. Therefore, further studies are needed to more fully explicate the possible relationship between fish consumption and rMSSD HRV.

Whereas most of the studies investigating the relationship between fish consumption and mental health have focused on mental health in general (the most used questionnaires are Center for Epidemiological Studies Depression Scale, Beck Depression Scale, and the WHO Well Being Index) [10], fewer studies have investigated the effect of fatty fish consumption on anxiety. Thus, due to the lack of dietary intervention studies, the aim of the present study was to investigate the effects of Atlantic salmon consumption on anxiety in a group of forensic inpatients with mental disorders. Since HRV and HR are regarded as biological and objective indices of emotion-regulation and anxiety, these biological indices were used as primary outcomes, while a self-report measure of anxiety was used as a secondary outcome measure. We expected that long-term fatty fish intervention would cause improvements in both the objective underlying biological mechanisms associated with anxiety and the subjective measure of anxiety. Moreover, rMSSD HRV, HR and self-reported anxiety were investigated in relation to vitamin D status, EPA and DHA.

## 2. Experimental Section

### 2.1. Study Sample

**Figure 1:**
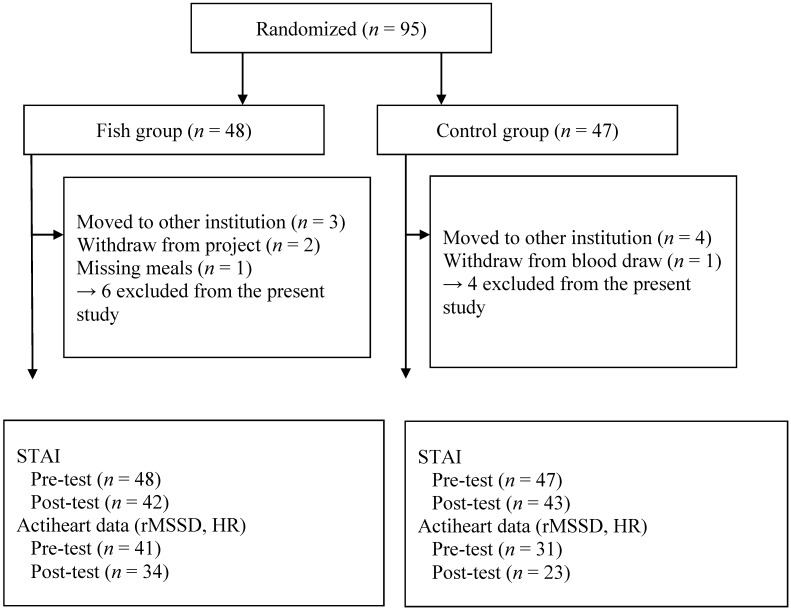
The flow diagram of the study progress.

Ninety-five sexual offenders residing at a secure forensic inpatient facility in the USA, with a mean age of 41 years (range: 21–60) were randomized into two groups. Those who wanted to participate were welcome, but IQ > 75 was used as an inclusion criteria. As illustrated in the flow diagram (Figure 1) 10 participants were lost due to different reasons. However, intention-to-treat analysis was not performed. Personality disorders (antisocial personality disorder, borderline personality disorder or personality disorder with antisocial traits) were diagnosed among 76% of the participants. Moreover, about 31% of the participants were diagnosed with some kind of anxiety disorder (GAD, OCD, PD or post-traumatic stress disorder) and about 18% were diagnosed with depression (major depressive disorder or depressive disorder). About 31% of the participants had both a personality disorder and an anxiety or depressive disorder.

Participants were matched on age, IQ and Psychopathy Checklist List-Revised score (PCL-R) [23] and then randomly assigned into the Fish group or the Control group. For randomization a computerized random number generator (in Excel) was used to assign each of the matched pairs. Thus, the random allocations to treatment or control groups were completed after all participants were enrolled and had completed baseline testing (pre-test battery). After participants provided informed consent, and following the completion of the pre-test battery, matched pairs were generated based on participants’ existing file data: age, IQ, and Psychopathy. Stratified Randomization was used to balance the treatment and control groups on age, IQ, and PCL-R score. The random allocation was done simultaneously for all subjects rather than sequentially and therefore it was not necessary to conceal any random allocation sequence. In terms of the matching one of the co-authors enrolled participants and another co-author produced the matched pairs (stratification). Then the same person who enrolled the participants set up the implementation of the computerized random number generator which determined the group to which each participant would be assigned.

*t*-Tests for independent samples were used in order to control for possible differences between the two groups on the IQ, PCL-R and BMI scores. *t*-Tests for independent samples showed that there were no differences between the groups in IQ score *t*(83) = −0.02, *p* < 0.98 or PCL-R score *t*(83) = −0.25), *p* < 0.80. Nor were there any differences between the groups in BMI at pre-test *t*(83) = −0.59, *p* < 0.56 or post-test *t*(73) = 1.61, *p* < 0.11 (see Table 1).

The flow diagram (Figure 1) presents a detailed description of the study progress. Heart rate signals were lost on 32 participants because of technical problems caused by the ECG electrodes (faulty electrodes). A post hoc power analysis reveals that based on the effect size of *d* = 0.69 (or medium *f* = 0.25), as found in the present study for the primary outcome measure (rMSSD HRV), and a total number of 57 participants and *p* < 0.05, the power in this study is 0.95.

### 2.2. Measures

Physiological activity was measured by recording heart rate (HR) and HRV using the Actiheart System (Cambridge Neurotechnology Ltd., Cambridge, UK) [24], a compact lightweight device that records HR and variability of R-R inter-beat intervals (IBI). The Actiheart clips onto a single ECG electrode (M-00-S/50 Blue Sensor) with a short ECG lead to another electrode that detects the ECG signal. The Actiheart was placed on the upper chest. Body mass index was calculated as weight in kilograms divided by the square of height in meters (kg/m^2^).

The State-Trait Anxiety Inventory (STAI) [25] was used to measure anxiety. The STAI is a well-validated self-report questionnaire [26]. Cronbach’s alpha was 0.916 and 0.917 in the current sample for the State and Trait dimensions, respectively.

### 2.3. Ethics and Procedure

The study protocol and all experimental procedures were approved by the Ethics Committee at the facility in Wisconsin, USA, and were in compliance with the Helsinki declaration. Participants were recruited by both written and oral information about the study. The participants had to sign an informed consent form and they were informed about their rights to withdraw from the study at any time for any reason without penalty. The participants were invited to participate in a project called “Nutrition and mental health” and they were informed that the purpose of the study was to investigate if nutrition (fatty fish or an alternative meal like chicken, pork, *etc.*) would have any effects on mental health.

Before (pre-test, July) and at the end of the intervention period (post-test, February) the participants went through a test procedure. Since the current study is part of a larger dietary intervention project the test procedure involved collecting a fasting blood sample, sleep data, exposure to experimental tasks, registering of psychophysiological activity, and completion of the STAI. For the present study five minutes of baseline (resting) HRV measured before and after the intervention were used as outcome measures. The baseline HR and HRV, measured as the root mean of the squared successive RR differences (rMSSD), was used for the present analyses. All participants were tested individually.

The Fish group received farmed Atlantic salmon (*Salmo salar* L.) for dinner, grilled or boiled (portion size of 150–300 g), three times per week for a period of 23 weeks (September–February). The Control group was provided an alternative meal (e.g., chicken, pork or beef), grilled or boiled, but with the same nutritional value as they normally received, three times per week during the same period. The groups were served the same side dishes (e.g., potatoes, different kinds of vegetables). Thus, all participants received some kind of intervention (fatty fish or different kinds of meat).

The inmates could go outdoor for recreation three times per day (max 2 h per time). We have not collected data on how much time the participants spent outside.

The production of the Atlantic salmon (*Salmo salar* L.) was at Skretting Fish Trials Station (Stavanger, Norway). The fish were harvested at about 4.5 kg and processed into skin and boneless portions (150 g), vacuum packed, and frozen (Rex Star Seafood, Tysnes, Norway).

The total fat and protein content of the salmon was 13.5 ± 3.3 and 20.3 ± 0.5 g/100-g fillet, respectively. The level of vitamin D was 5 ± 3 µg/100-g fillet. The intake of EPA+DHA and vitamin D in a portion (300 g) of Atlantic salmon were 4.8 g and 15 μg, respectively. The standard portion was 300 g three times a week; however during the final four- weeks of the study, they were served portion sizes of 150 g of salmon. The content of several undesirable substances was also determined in the Atlantic salmon. The level of mercury was 22 µg/kg, and the level of dioxins and dioxin-like PCBs was 0.48 ng TEQ/kg; both are far below the EUs upper limits of 500 µg/kg and 6.5 ng TEQ/kg in fish, respectively. Taking into account the amount of salmon consumed per week during the weeks with the highest salmon intake, the intake of dioxin and dioxin-like PCBs per week represents 31% of the tolerable weekly intake (TWI) in a person weighing 100 kg [27]. Persons with higher body weight will have a correspondingly lower percentage of TWI. Importantly, no adverse side effects were reported during the intervention trial [12].

For analyses of the psychophysiological data raw data from the Actiheart system was imported to Statistica by means of a self-written MATLAB (The Math Works, Natick, MA) program. HRV was measured as the rMSSD of the inter-beat interval (IBI). rMSSD is a time domain measure of HRV and reflects parasympathetic activity [28]. The rMSSD data were log transformed prior to analysis [28].

### 2.4. Statistical Analysis

Differences between the two groups on measures of cardiovascular reactivity (rMSSD and HR) and state- and trait-anxiety were analysed by two-way repeated measures ANOVAs ((Fish *vs.* Control group) × 2 (pre- *vs.* post-test conditions)). The dependent variables were rMSSD, HR, state- and trait-anxiety. The analyses were followed up by Bonferroni correction. In order to examine the magnitude of the significant differences between the independent means we calculated the effect sizes as Cohen’s *d* [29]. Pearson Product Moment correlation analyses were used in order to investigate the relationship between mental health variables (cardiac parameters; rMSSD, HR, and state- and trait-anxiety) and specific nutrients (vitamin D status, EPA and DHA).

## 3. Results

### 3.1. Descriptive Statistics

Means and standard deviations for all related variables age, IQ, PCL-R, EPA, DHA, state-and trait-anxiety, rMSSD and HR during pre- and post-test are presented in Table 1. Also the numbers of participants in each group with personality disorders, anxiety and depression related diagnoses are presented in the table. Means and standard deviations for vitamin D measured as 25 hydroxy vitamin D (25 OHD) are reported previously. Fish group pre-test: 85 ± 36 nmol/L and post-test: 71 ± 27 nmol/L; Control group pre-test: 75 ± 34 nmol/L and post-test 55 ± 23 nmol/L [12].

**Table 1:** Shows the number of participants with personality disorders (PD), anxiety disorders (AD) and depression disorders (DD) in each group, the means and standard deviations for the age, IQ, PCL-R, EPA, DHA, state- and trait-anxiety, the root mean square of successive differences (rMSSD), and heart rate (HR), for both groups during pre- and post-test. The rMSSD data are log-transformed.

Clinical Characteristics	Control Group (*n* = 43)			Fish Group (*n* = 42)		
	Pre-test	Post-test	*p*	Pre-test	Post-test	*p*
PD	39% (*n* = 33)			38% (*n* = 32)		
AD	14% (*n* = 12)			16% (*n* = 14)		
DD	11% (*n* = 9)			7% (*n* = 6)		
PD+AD/DD	19% (*n* = 9)			12% (*n* = 10)		
**Age**	40.84 ± 9.14			42.76 ± 9.72		0.35
**IQ**	95.84 ± 13.02			95.79 ± 11.10		0.98
**PCL-R**	25.64 ± 5.58			25.30 ± 7.03		0.80
**BMI**	32 ± 13 ^1^	29 ± 7 ^2^		30 ± 5 ^1^	31 ± 5 ^2^	^1^ 0.56 ^2^ 0.11
**EPA**	11 ± 8	17 *±* 16 ^2^	0.02	10 *±* 6	31 *±* 12 ^2^	0.001 ^2^ 0.001
**DHA**	79 *±* 34	88 *±* 47 ^2^	1	73 *±* 25	158 *±* 50 ^2^	0.001, ^2^ 0.001
**Anxiety**						
State-Anxiety	32.37 *±* 10.24	32.42 *±* 9.98	0.97	33.71 *±* 9.70	29.79 *±* 8.42	0.006
Trait-Anxiety	38.14 *±* 10.10	36.63 *±* 8.58	0.16	37.60 *±* 8.23	36.93 *±* 8.44	0.54
**Psychology**		**(*n* = 23)**			**(*n* = 34)**	
rMSSD	1.48 *±* 0.31	1.39 *±* 0.33	0.18	1.38 *±* 0.26	1.57 *±* 0.29	0.001
HR	73 *±* 12	78 *±* 11	0.03	76 *±* 12	72 *±* 12	0.01

^1^ between group differences at pre-test. ^2^ between group differences at post-test. The sum EPA + DHA ANOVA results have been reported in Hansen *et al.* [12].

### 3.2. Fatty Fish Consumption and Psychophysiology

**Figure 2:**
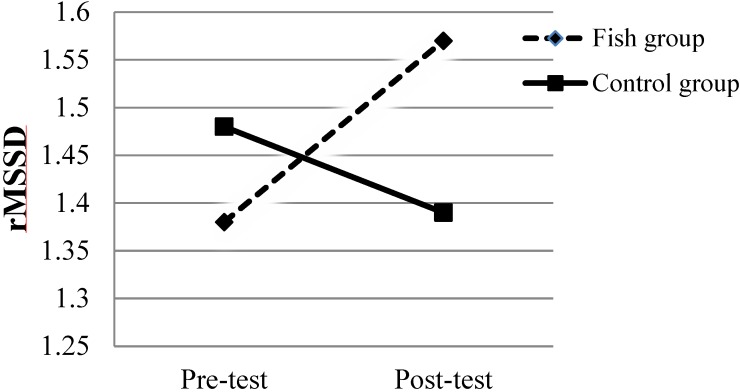
The root mean square of successive differences (rMSSD) for both groups during pre- and post-test. The standard deviations are presented in Table 1.

For the rMSSD data the two-way ANOVA showed no main effect of groups *F*(1,55) = 0.39, *p* = 0.536, η^2^ = 0.014. There was no significant effect of time, *F*(1,55) = 1.26, *p* = 0.266, η^2^ = 0.02. However, there was a significant interaction effect between groups and time, *F*(1,55) = 10.26, *p* = 0.002, η^2^= 0.15. In follow-up tests with Bonferroni correction, the results revealed that the Fish group had a significant increase in rMSSD from pre- to post-test (*p* < 0.007; *d* = 0.69; see Figure 2).

In line with the rMSSD data the HR data showed no significant effect of groups *F*(1,55) = 0.17, *p* = 0.679, η^2^ = 0.2 or time *F*(1,55) = 0.04, *p* = 0.838, η^2^ = 0.0006. However, there was a significant interaction effect between the groups and time *F*(1,55) = 18.34, *p* = 0.001, η^2^ = 0.25. Bonferroni correction demonstrated that the Fish group had a significant decrease in HR from pre- to post-test (*p* < 0.01; *d* = 0.45), while the Control group had a significant increase in HR from pre- to post-test (*p* < 0.03; *d* = 0.39; see Table 1).

### 3.3. Fatty Fish Consumption and Anxiety

**Figure 3:**
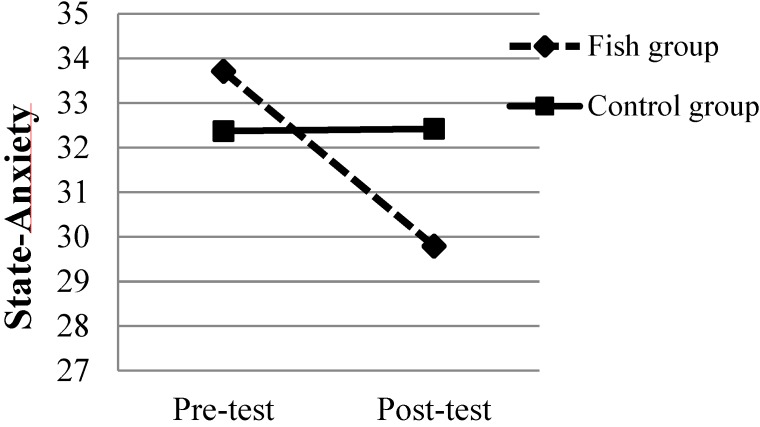
The level of state-anxiety for both groups during pre- and post-test. The standard deviations are presented in Table 1.

For the state-anxiety scale there was no main effect of groups, *F*(1,83) = 0.122, *p* = 0.728, η^2^ = 0.005. However, there was a main effect of time (pre- and post-test conditions) *F*(1,83) = 3.99, *p* = 0.049, η^2^ = 0.44 showing lower levels of state-anxiety at post-test compared to pre-test (*p* < 0.05; *d* = 0.20). Finally the interaction between the groups and time was significant *F*(1,83) = 4.18, *p* = 0.043, η^2^ = 0.46. In follow up tests with Bonferroni correction, the results revealed that the Fish group had a significant decrease in state-anxiety from pre- to post-test (*p* < 0.03; *d* = 0.43; see Figure 3). This was not found in the Control group. For trait-anxiety no significant results were found.

### 3.4. Correlations between HRV Parameters and Specific Nutrients

At pre-test no significant correlations were found. However, at post-test the correlation analyses demonstrated that vitamin D status was significantly and positively related to rMSSD (*r* = 0.27, *p* < 0.05). No other relationships were found at post-test.

### 3.5. Correlations between Anxiety and Specific Nutrients

Pearson product moment correlations revealed no significant relationship between state- or trait-anxiety and vitamin D status at pre-test. The results revealed that there was a positive relationship between EPA and state-anxiety (*r* = 0.31, *p* = 0.005) before the intervention. However, this pattern of relationship was not evident at the end of the intervention (*r* = −0.06, *p* = 0.610). No other significant relationships were found.

## 4. Discussion

Overall, the present findings showed that Atlantic salmon consumption caused improvements in both HRV and HR, as well as reductions in state-anxiety in a sample of male forensic inpatients. Interestingly the results also demonstrated a relationship between vitamin D status and rMSSD at post-test even though neither of the groups was vitamin D deficient at any time point. However, the Control group had a suboptimal vitamin D status (for US population: <75 nmol/L) [30] at post-test, while the vitamin D status in the Fish group was still close to the optimal level at post-test as described more thoroughly in Hansen *et al.* [12]. At pre-test there was a positive relationship between EPA and state-anxiety. However, this trend was not observed at post-test.

The present results showed that fatty fish consumption caused changes in HRV which is regarded as an essential underlying biological mechanism involved in anxiety and emotion-regulation [16].Interestingly, the present long-term dietary fish intervention study was in line with Mozaffarian *et al.* [13] who found a relationship between fish consumption and specific HRV components such as the rMSSD. Additionally, the present 23 week study helps to clarify the finding of a previous investigation [22] demonstrating that a fish intervention of only eight weeks was not sufficient to cause any change in rMSSD. Thus, longer interventions may be necessary to produce reliable effects.

Importantly, the result from the self-reported anxiety measure was in line with the biological objective measure of anxiety. The current results demonstrated that Atlantic salmon consumption caused a significant decrease in state-anxiety, but not trait-anxiety. This extends previous research investigating the relationship between fish consumption and depression [5]. The state-anxiety is characterized by physiological arousal [31] and it has been argued that the state-anxiety scale involves elements of both emotional-autonomic activation and cognitive worry [32]. The STAI is a well validated instrument and previous studies have also demonstrated that the state-anxiety scale is sensitive to interventions such as a single session of slow breathing [33]. Thus, the present results suggest that state-anxiety may also be sensitive to nutritious food intervention. The lack of a significant reduction in the trait-anxiety scale in the present study may be because trait-anxiety is associated with more stable personality traits and a stable individual vulnerability to experience anxiety [25]. As such, trait-anxiety may be more difficult to change during a 6 month intervention study.

Another interesting finding from the current study was that the Control group revealed a significant increase in HR from pre- to post-test. Based on these changes in HR in the Control group, one could speculate whether the autonomic nervous system (ANS) is sensitive to seasonal changes of vitamin D status [11,34], due to a relationship between vitamin D status and ANS function. For example, both may be regulated by the same underlying biomarker/mechanisms such that as vitamin D status decreases during the winter due to reduced sun exposure, HR increases due to reduced vagal inhibition. It is worth noting that all participants had the possibility of outdoor activities at the time of the year when the UV light is sufficient for skin vitamin D synthesis [35]. Importantly the present study demonstrated that there was a relationship between vitamin D status and HRV.

It has earlier been speculated whether a beneficial effect of fish consumption on HRV could be due to the improved vitamin D status and a possible association with serotonin [36]. The speculation was based on the argument that vitamin D is a key factor in the regulation of serotonin [37], which in turn is very important for the regulation of HRV [38]. As suggested by Kellett *et al.* [39] serotonin contributes to reflex activation of parasympathetic outflow, *i.e.*, increased HRV. The present study does not allow us to draw conclusions about which of these possible relationships holds. However, future studies could include measures of serotonin in order to get a deeper understanding of the exact mechanisms involved in the beneficial effects of fatty fish consumption.

At pre-test the level of EPA correlated positively with state-anxiety. However, this relationship was not observed during post-test. Overall our results suggest beneficial effects of fatty fish consumption, but these effects do not seem to be explained by the marine fatty acids as suggested in other studies focusing on mental health [5,6]. As shown in Table 1 the Fish group had a significant increase in both EPA and DHA from pre- to post-test. Also the mean values of EPA increased from pre- to post-test in the Control group (*d* = 0.47). However, the increase in EPA was much larger in the Fish group (*d* = 2.21), and at post-test the Fish group had a significantly higher level of EPA compared to the Control group (*d* = 0.99). Thus, it is important to be aware that the beneficial effect of Atlantic salmon consumption observed in the present study may be due to multiple mechanisms. In addition to its content of marine omega-3 fatty acids and vitamin D, seafood is also a unique source of other nutrients like selenium, iodine, vitamin B_12_ and high quality proteins [40]. The beneficial effect of the present intervention on state-anxiety and HRV might therefore be attributed to several of these additional nutrients or the salmon as whole food compared with the diet of the control group.

There are also some limitations with this study that should be mentioned. First, the use of a sample of only adult male forensic inpatients may make it difficult to generalize the results to other groups in the population. Future studies should investigate the relationship between fatty fish consumption and mental health related variables in other populations (e.g., in non-forensic samples, females, children and adolescents). Second, the STAI is a well validated self-report questionnaire and widely used in order to measure anxiety. It also correlates with depression, and it can be regarded as a measure of general distress and mental health [41]. One could question whether state-anxiety is a pure measure of anxiety or whether it is a general measure of mental health. Third, blinding is not possible when it comes to food interventions, and no one was blinded in this study. The participants may have been influenced by their knowledge about the food they were served, the fatty fish or, e.g., the chicken. However, chicken is generally regarded as healthy food as well due to the low level of fat and essential nutrients. Thus, both groups could have been influenced by the food they were served. In order to gain more knowledge about the beneficial effect of fatty fish consumption, dietary intervention is necessary. The use of both a self-report measure of anxiety along with an objective biological measure of anxiety, minimizes the likelihood that the results were caused by the participants’ expectations due to knowledge of the health benefit of fatty fish consumption.

## 5. Conclusions

In summary, the present study demonstrated that Atlantic salmon consumption had beneficial effects on HRV measured as rMSSD, HR and state-anxiety. If this is solely a vitamin D effect, a routine vitamin D supplementation could be adequate. However, fatty fish contributes several other essential nutrients such as marine omega-3, iodine, selenium, and proteins, and increased seafood consumption may also substitute for other meals with a lower nutritional value. Further investigation is therefore required for an understanding of the precise mechanism(s) responsible for the beneficial effects of Atlantic salmon reported here.
